# Professor Wilbur Julio Kraak - a tribute

**DOI:** 10.17159/2078-516X/2024/v36i1a19878

**Published:** 2024-08-15

**Authors:** Heinrich Grobbelaar

**Affiliations:** Associate Professor of Sport Psychology in the Division of Sport Science, Department of Exercise, Sport and Lifestyle Medicine, Faculty of Medicine and Health Sciences, Stellenbosch University, South Africa

[Fig f1-2078-516x-36-v36i1a19878]

**Figure f1-2078-516x-36-v36i1a19878:**
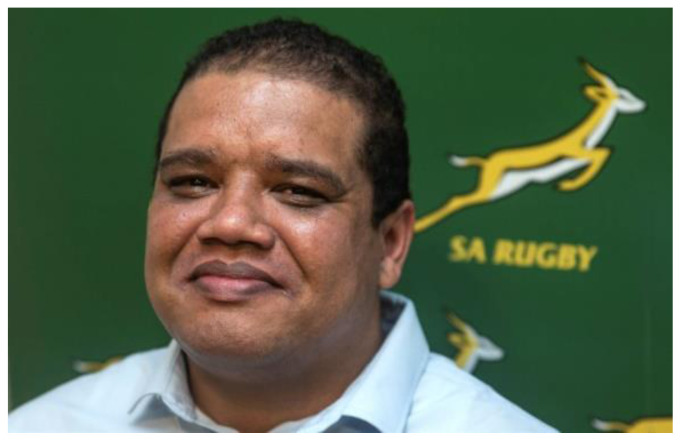

Saturday, July 27, 2024, saw the sudden passing of my former student, colleague, previous line manager, and close friend, Professor Wilbur Julio Kraak. Wilbur suffered a stroke during a lecture to University of the Western Cape (UWC) students on Friday, July 26. We spoke on the phone just days before, planning to meet in person the following week and attend the Spring Sport Science Summit in Potchefstroom in September.

Wilbur was born on 25 April 1984 and grew up in the Western Cape town of Mamre, the oldest child of Wilfred and Alna Kraak, with sister Tammy, born a couple of years later. Wilbur was head boy of Mamre Primary School, where the first of three memorial services were recently held in his honour. Wilbur was a talented rugby player and enrolled for a Diploma in Coaching Science (specialising in Rugby) at the then Potchefstroom University for Christian Higher Education (now North-West University – NWU) in 2002, because he did not have full matric exemption. Our paths first crossed in his first year, in the “LEER111” module, aimed at facilitating first-year students’ transition into university and equipping them with basic learning, time management and coping skills to flourish in their studies and life. I was his lecturer and still recall how he and his closest friends sat at the back of the class. They had tons of energy and struggled to sit still. He completed the diploma program as a final-year student in 2004 and gained entry to the BA program in Human Movement Science and Psychology (2005 – 2006). During his undergraduate studies, he was a proud resident of “Overs”, serving on their House Committee and on the NWU Student Representative Council (Sport and Recreation portfolio).

Our paths crossed again during his BA Hons Sport Science years (2007 – 2008), both in the classroom and on the rugby field. This time as a rugby referee, having traded his rugby boots for a whistle. Andre Watson, the most capped international referee at the time, rated him highly as an upcoming referee. I didn’t… because he always penalised my players, and I thought they were little angels who never made mistakes. But Wilbur blew his whistle on them and firmly stood by his decisions. I recall a Friday evening match at the Fanie du Toit sports ground when a lightning bolt struck the floodlights of the nearby cricket field. I shouted: “We need to get off now”, knowing that he would seldom have encountered lightning growing up in the Western Cape. When he blew the whistle for the players to get off the field, some were already sitting in the changeroom. Another funny incident during a match between the Leopards and Blue Bulls u/19 teams at Olën Park Stadium in Potchefstroom in which Wilbur was the linesman, was when the goal posts fell over after the home team held up an attacking maul just before halftime. We often chatted about the incident and even watched the video clip, laughing about the record-breaking speed with which some of the players (with whom he still had contact more than a decade later) ran out of harm’s way.

Wilbur was a hard-working student in my Hons sport psychology module during my final year as a lecturer at the NWU. My parting gift to him, when I moved to Stellenbosch University (SU), was the one-minute manager book entitled “Who Moved My Cheese?” The life lesson in this story is the need to evaluate situations constantly and to move on when the cheese (a metaphor for what is important in life) becomes less stale or is taken from you. He certainly applied this life lesson in the years to come, constantly challenging his status quo.

Refereeing took its toll on this “peoples person”, because it was such a “lonely job.” He explained that whilst one team was celebrating in the changeroom, their opponents were often upset with him, and he mostly ended up sitting alone in his hotel room after officiating a match far from home. He completed his M.A. in Sport Science at the NWU (2009 – 2011) on movement patterns and heart rate recordings of rugby referees during match play. His supervisor was the late Professor Dawie Malan, who also happened to be my PhD promotor around that time. Wilbur embraced the emerging sports science field of performance analysis and soon started doing match analysis for the NWU-PUK Rugby Institute and the Leopards senior provincial teams. Next, he started coaching his residence team, much to my dismay, because they snuck into the playoffs and went on to beat my old residence in the final. His experience as a player, referee, and performance analyst would serve him well as a coach.

In 2010, he was headhunted for a Junior Lecturer position in SU’s Department of Sport Science to head up the third-year coaching specialisation course. During the next 14 years, he presented modules in coaching specialisation, performance analysis, peak performance, sports management and rugby practical. He obtained his PhD in 2015 under the supervision of Professors Ranel Venter (US) and Derik Coetzee (University of the Free State, UFS), entitled “The effect of law changes on the match profile of international and national rugby union between 2007 and 2013.” Prof Coetzee became his mentor through the SU Early Career Academic Development (ECAD) programme, and Wilbur later mentored many of the new SU academics. At the time of his passing, Wilbur held an extraordinary professorship at UFS and lectured part-time to SU Sport Science students.

In 2020, Wilbur was promoted to Associate Professor, skipping the rank of Senior Lecturer, based on his exceptional research outputs and overall strong academic CV. This academic “non-starter’s” career had taken off. He has successfully supervised dozens of postgraduate students. Their research focused on sports performance analysis, injury risk reduction in team sports and coach education. Many of his students commented that he coached rather than supervised them, allowing them to develop as researchers and express themselves freely. He has 43 publications in peer-reviewed journals to date, with more than 500 citations and an h-index of 16. He previously held the National Research Foundation Yrating for young researchers and was awaiting the outcome of his new rating application at the time of his passing. His publication and citation numbers will grow, as he has multiple papers currently under review and was supervising at least 10 postgraduate students at the time of his passing. My sincere condolences go out to his current and former students.

It was the field of coaching and coach education where Wilbur was to make his biggest contribution as World Rugby Level 3 coach and World Rugby Coach Educator. He completed SA Rugby’s Elite Coaches programme in 2023. He hosted and presented at SU’s Sports Coaching Summit annually, with around 250 community, club, and elite-level coaches attending. He was the coach education lead of the Commonwealth Games’ GAPS initiative for para-athletes and coaches in track-and-field, table tennis and powerlifting from 13 African countries. He is the technical expert on eTV’s Bôll & Ôll Rugby show. He was a Director of the International Society of Performance Analysis of Sport (ISPAS) with a Level 5 Performance Analyst grading. He founded WJK Coaching Consultancy, which will continue striving to be the leader in developing and providing services to coaches, specialist coaches and sports administrators. He coached teams like St George’s Rugby Football Club (RFC), Schotsekloof Walmers RFC, NTK RFC, and Maties Rugby Club, all competing in the Western Province A-league.

He offered rugby coaching clinics to the inmates at Drakenstein Correctional Centre, believing that sport offered a healthy outlet and hope for these players. He was vocal about creating coaching opportunities for Black coaches and administrators in South Africa. Wilbur completed two terms as Resident Head of Majuba Men’s Hostel at SU, and was much liked and respected by his students, players, colleagues and mentees.

Following the restructuring of the Department of Sports Science and the establishment of the Department of Exercise, Sports and Lifestyle Medicine, he fulfilled the role of Head of the Division of Sports Science (2022 – 2023). His colleagues will remember his people-oriented and light-hearted approach and miss his frequent check-ins to see how we were doing. On 1 August 2023 Wilbur started as Professor of Sports Science at the University of the Western Cape (UWC). A remarkable journey from academic “non-starter” to Professor in 21 years. In his first year at UWC, he left his mark and leaves a big hole.

On behalf of SU’s Department of Exercise, Sport and Lifestyle Medicine and the South African Sports Medicine Association, we would like to extend our sincerest condolences to his wife Mirinthea, children Wilrique and Zhea, his parents, sister, extended family members and friends. Wilbur will be missed as a person, educator, scientist, mentor, coach and dear friend.

